# Cold Exposure Induces Intestinal Barrier Damage and Endoplasmic Reticulum Stress in the Colon *via* the SIRT1/Nrf2 Signaling Pathway

**DOI:** 10.3389/fphys.2022.822348

**Published:** 2022-04-20

**Authors:** Jingru Guo, Huijie Hu, Zhuo Chen, Jing Xu, Junshu Nie, Jingjing Lu, Li Ma, Hong Ji, Jianbin Yuan, Bin Xu

**Affiliations:** National Experimental Teaching Demonstration Center of Animal Medicine Foundation, College of Animal Science and Veterinary Medicine, Heilongjiang Bayi Agricultural University, Daqing, China

**Keywords:** cold exposure, colon, tight junction, endoplasmic reticulum stress, SIRT1, nrf2

## Abstract

Ambient air temperature is a key factor affecting human health. Long-term exposure to a cold environment can cause various diseases, while the impact on the intestine, the organ which has the largest contact area with the external environment, cannot be ignored. In this study, we investigated the effect of chronic cold exposure on the colon and its preliminary mechanism of action. Mice were exposed to 4°C for 3 hours a day for 10 days. We found that cold exposure damaged the morphology and structure of the colon, destroyed the tight junctions of the colonic epithelial tissue, and promoted inflammation of the colon. At the same time, cold exposure also activated the unfolded protein response (UPR) in the colon and promoted apoptosis in intestinal epithelial cells. Chronic cold exposure induced oxidative stress *in vivo*, but also significantly enhanced the response of the Nrf2 pathway that promotes an anti-oxidant effect. Furthermore, we demonstrated that chronic cold exposure promoted p65 acetylation to aggravate the inflammatory response by inhibiting SIRT1. Similar results were observed following SIRT1 knock-down by shRNA in Caco-2 cells treated with Thapsigargin (Tg). Knock-down of SIRT1 promoted nuclear localization of Nrf2, and increased the level of Nrf2 acetylation. Taken together, our study indicates that cold exposure may aggravate endoplasmic reticulum stress and damage epithelial tight junctions in the colon by inhibiting SIRT1, which promotes nuclear localization of Nrf2 and induces an anti-oxidant response to maintain intestinal homeostasis. These findings suggest that SIRT1 is a potential target for regulating intestinal health under cold exposure conditions.

## Introduction

The impact of a low temperature environment on human health is significant. Zhang et al. found that moderate cold temperature contributed to a significant amount of the total health burden (Zhang et al., 2020a). For example, cold exposure can cause reproductive organ dysfunction, abnormal glucose metabolism, neurotransmitter disorders, and promote neuroinflammation (Yao et al., 2018; Xu et al., 2019; Wang et al., 2020). In addition, cold exposure can increase disease sensitivity, such as promoting bone loss, and increasing the risk of cancer by regulating the expression of transcription factors (Bandyopadhayaya et al., 2020; Singh et al., 2020).

Intestinal diseases, such as diarrhea, stress-induced bowel disease and inflammatory bowel disease (IBD), are particularly prevalent in cold environments. The colon is a barrier tissue, which can, to a certain extent, regulate and defend against changes in the external environment (Evans and Corfe, 2021). However, since the colon has the highest abundance of intestinal flora, once this barrier is damaged, toxins and bacteria can leak into the intestinal tissues, increasing the sensitivity of the body to enteritis and causing intestinal inflammation (Öhman et al., 2015; Pellissier and Bonaz, 2017). Thus, the integrity of healthy tight junctions between epithelial cells in the colon is crucial to the stability of the colon barrier function.

Previously, the pathogenesis of enteritis has been shown to be closely related to the unfolded protein response (UPR) of intestinal epithelial cells (IECs) (Hosomi et al., 2015; Ma et al., 2017). IECs from IBD patients have been shown to exhibit an excessive induction of endoplasmic reticulum (ER) stress linked to altered intestinal barrier function and inflammation (Solà Tapias et al., 2021; Wang et al., 2021). Li et al. found that knock-down of inositol-requiring enzyme 1α (IRE1α) can inhibit the growth of heterogeneous tumors *in vivo* and colon cancer cells *in vitro* (Li et al., 2017). Furthermore, cold exposure can lead to abnormalities in the neuro-endocrine-immune network, as well as dramatic changes in blood glucose and blood lipid concentrations, which may activate the UPR of cells (Lian et al., 2019). Thus, we speculate that ER stress plays a key role in cold-induced intestinal barrier damage.

Recent studies have shown that sirtuin 1 (SIRT1) plays a key role in the development of various diseases related to ER stress, including IBD (Deuring et al., 2014; Melhem et al., 2016). SIRT1 belongs to the sirtuin family of nicotinamide adenine dinucleotide (NAD+) dependent deacetylases, which can interact with or deacetylate various signaling molecules, transcription factors, histones and non-histone proteins (Chandramowlishwaran et al., 2020). SIRT1 has been shown to improve ER stress-induced cell death by deacetylation of eukaryotic translation initiation factor 2α (eIF2α) and X-box binding protein1 (XBP-1) (Prola et al., 2017; Chou et al., 2019). However, the role of SIRT1 in cold-induced stress has not been examined.

In addition, activation of UPR during oxidative stress is an adaptive mechanism for maintaining cell function and survival. In many ER stress-related models, ER stress and oxidative stress accentuate each other in a positive feedforward cycle, which interferes with cell function and activates apoptosis-promoting signal transduction pathways (Malhotra and Kaufman, 2007). ER stress and oxidative stress have also been observed in a variety of enteritis (Almenier et al., 2012; Kaser et al., 2013). Nrf2 is a transcription factor that plays a central role in the intestinal antioxidant stress response, which is activated by ER stress and oxidative stress (Wu et al., 2021). Under conditions of ER stress, Nrf2 is phosphorylated, and once activated, localizes to the nucleus where it induces antioxidant stress response genes for cellular protection (Cullinan and Diehl, 2006).

Previously, we have shown that cold exposure induced oxidative stress and activated the antioxidant pathway in liver. Here, we explored the impact of exposure to external low temperatures on the colon, as well as examine the underlying mechanisms of action of SIRT1.

## Materials and Methods

### Animals and Animal Model

Five-week-old male C57BL/6 mice (body weight 18–20 g) were purchased from Changsheng (Changchun, China) and randomly divided into two groups (*n* = 6). All mice were placed in a phytotron for 7 days to adapt before grouping. The cold group mice were exposed to 4°C for random 3 h between 8:00–20:00, and placed in the phytotron at 24 ± 2°C at other times. The control group was placed in the phytotron at 24 ± 2°C for the entirety of the experiment. All mice followed a 12 h (8:00–20:00) light-dark cycle with a humidity of 40 ± 5% and ad libitum access to food and water. All mice were anesthetized with ether after treatment and euthanized by cervical dislocation. Colon tissue was collected, and either fixed or frozen at −80°C after the tissue length had been recorded.

### Immunofluorescence, TUNEL and Hematoxylin-Eosin Staining

Colon tissue was fixed in 4% paraformaldehyde (BL539A, Biosharp, China), dehydrated and cleared using ethanol and xylene. The transparent tissue block was immersed in melted paraffin, and cut it into 5–8 µm slices after the paraffin had solidified.

After dewaxing and hydration, immunofluorescence staining was carried out on tissue slices by incubating the slices with 0.2% TritonX-100 for 15 min, blocking for 2 h, then incubating with primary antibodies against ZO-1 (#21773-1-AP, Proteintech, 1:200) overnight at 4°C. Samples were incubated with fluorescein-conjugated secondary antibody for 1 h at 37°C, and treated with anti-fluorescence quenching sealing solution (containing DAPI). Finally, tissue slices were photographed using a laser scanning confocal microscope.

After dewaxing and hydration, TUNEL staining was performed by incubating tissue slices with 20 μg/ml DNase-free proteinase K at 20–37°C for 15–30 min. After washing, samples were incubated with 50 μL TUNEL detection solution at 37°C for 1 h, then photographed using a laser scanning confocal microscope.

After dewaxing and hydration, H&E staining was carried out by dying tissue slices with hematoxylin staining solution and eosin staining solution. Samples were then blocked with neutral gum and photographed using a light microscope.

### Transmission Electron Microscope

Fresh colon tissue was fixed in 2.5% glutaraldehyde and dehydrated with ethanol. The treated tissue was embedded in epoxy resin, and cut into 70 nm slices using an ultramicrotome. After staining with uranium acetate and lead citrate, morphological changes in the ER were observed and photographed using a JEM SX 100 TEM.

### Biochemical Analysis

Levels of superoxide dismutase (SOD) activity, glutathione (GSH) and malondialdehyde (MDA) were measured in the colon tissue using specific kits (#S0101S, #S0053, #S0131S, Beyotime, China) according to the manufacturer’s instructions.

### SIRT1 Activity

Total protein was extracted from the colon tissue and the level of SIRT1 activity was tested using a SIRT1 Assay Kit (#CS1040, Sigma-Aldrich, United States) according to the manufacturer’s instructions.

### Cell Culture and Treatment

Caco-2 cells were cultured in DMEM medium containing 10% fetal bovine serum at 37°C in an atmosphere of 5% CO_2_. Cells were passaged 2–3 times a week.

An ER stress model was constructed by stimulating Caco-2 cells with 1 µM Thapsigargin (Tg) for 12 h. The SIRT1-shRNA sequence (5′-GCC​ATG​TTT​GAT​ATT​GAG​TAT-3′) was synthesized by Hesheng SyngenTech (China) and used to knock down SIRT1 expression in Caco-2 cells. The following transfection conditions were used: 2.5 μg shRNA plasmid and 4 μL Lipo8000 transfection reagent were added to six-well plates and samples were collected after 48 h.

### Immunofluorescence and Hoechst Staining

Caco-2 cells were cultured on slides, treated with Tg and transfected with shRNA. Slides were incubated with 0.2% TritonX-100 for 15 min, blocked for 2 h and immunofluorescent staining was carried out as described earlier. The expression and distribution of ZO-1 was observed and photographed using a laser scanning confocal microscope. The Hoechst Staining Kit (#RG027, Beyotime, China) was used to stain the nuclei.

### Western Blot Analysis

After treatment, colon tissue or Caco-2 cells were lysed with RIPA lysis buffer containing 1% PMSF. Total protein and nucleoprotein were extracted using the RIPA Lysis Buffer (#P0013C, Beyotime, China) and Nuclear and Cytoplasmic Protein Extraction Kit (#P0028, Beyotime, China), and the protein concentration was determined using a BCA Protein Assay Kit (#P0010, Beyotime, China) according to the manufacturer’s instructions. Protein samples were separated by SDS-PAGE and transferred to a PVDF membrane. After blocking, the membrane was incubated in primary antibodies overnight at 4°C. After washing, samples were incubated with secondary antibodies (#SA00001-2and#SA00001-1, Proteintech, 1:10,000). Protein bands were visualized using an ECL luminescence agent and chemiluminescence detector. The following primary antibodies were used: ZO-1 (21773-1-AP, Proteintech, 1:7,000), Occludin (27260-1-AP, Proteintech, 1:3,000), Claudin-1 (13050-1-AP, Proteintech, 1:5,000), TNFα (#17590-1-AP, Proteintech, 1:1,000), IL-1β(26048-1-AP, Proteintech, 1:800), IL-6 (66,146-1-lg, Proteintech, 1:1,000), GRP78 (11587-1-AP, Proteintech, 1:6,000), XBP-1 (24868-1-AP, Proteintech, 1:1,000), CHOP(15204-1-AP, Proteintech, 1:1,000), eIf2α (#A0764,ABclonal, 1:1,000), p-eIf2α (#AP0341, ABclonal, 1:2000), Bax (#50599-2-lg, Proteintech, 1:10,000), Bcl-2 (#60178-1-lg, Proteintech, 1:3,000), Caspase3 (66,470-2-lg, Proteintech, 1:1,000), Cytochrome C (10993-1-AP, Proteintech, 1:5,000),SIRT1(#13161-1-AP, Proteintech, 1:500) Nrf2(16396-1-AP, Proteintech, 1:1,000), Keap1 (10503-2-AP,Proteintech, 1:3,000), NQO1 (11451-1-AP, Proteintech, 1:1,000), HO-1 (10701-1-AP, Proteintech, 1:3,000), p65 (#10745-1-AP, Proteintech, 1:2000), Actin (66009-1-Ig, Proteintech, 1:10,000), Lamin B (12987-1-AP, Proteintech, 1:5,000).

### Co-Immunoprecipitation Assays

Magnetic dynabeads were suspended in 200 µL binding and washing solution containing 2 µg Nrf2 antibodies. The beads were incubated for 10 min at room temperature with shaking and placed on the magnet. The dynabead-antibody complex was washed with 200 µL of antibody binding and washing buffer. Next, 500 µL of the total colon tissue or Caco-2 cell homogenates were added to each of these tubes (containing Nrf2 antibody), mixed and incubated for 10 min at room temperature. All tubes were placed on the magnet and the supernatants were removed. After washing, 100 dynabead-antibody-antigen complexes of each sample were suspended in 100 µL washing buffer and mixed gently. The antigens were eluted using 200 µL eluent buffer and incubated for 2 min at room temperature. The supernatants were removed and placed into clean tubes, and western blotting was carried out to detect the acetylated form of Nrf2.

### Dual Luciferase Reporter Gene Assay

The dual luciferase reporter assay was performed in Caco-2 cells that had been transfected with shRNA and treated with Tg treatment. The cell culture medium was replaced by a serum-free medium when the density reached 70%. The pGL4.37 (luc2P/ARE/Hygro) plasmid was transfected for 12 h, and the serum-free medium was converted to a complete medium. After 24 h of transfection, the detection preparation was completed as per the instructions stipulated in the dual-luciferase reporter gene detection kit (#RG027, Beyotime, China). The fluorescence intensity was measured by a multi-functional enzyme labeling instrument, and the activity of the Nrf2 promoter was calculated.

### Statistical Analysis

All statistical parameters were calculated using GraphPad Prism 8 (GraphPad) and Excel software. Differences were analyzed using t-tests (for two groups) and one-way ANOVA for multiple comparisons. *p* < 0.05 was considered statistically significant. Values are expressed as mean ± standard error of the mean (SEM), *p < 0.05, **p < 0.01, ***p < 0.001.

## Results

### Cold Exposure Induces Tight Junction Damage and Promotes Inflammation in Mice Colon

**FIGURE 1:**
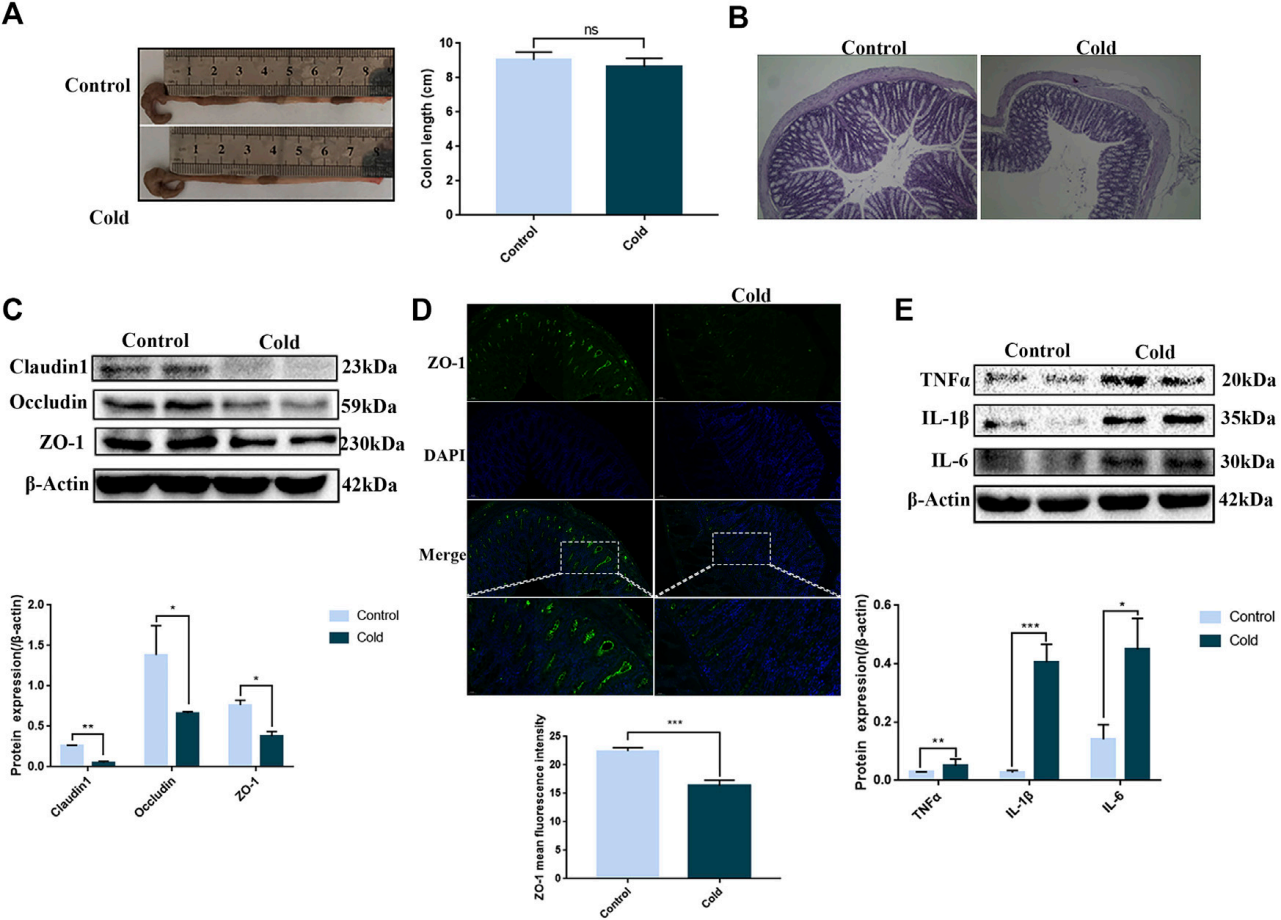
Cold exposure induces tight junction damage in the colon and promotes colonic inflammation. Effects of cold exposure on mouse colon length **(A)** and morphology by H&E staining (×100) **(B)**. Effects of cold exposure on the protein expression levels of claudin-1, occludin and ZO-1 were assessed by western blot analysis **(C)** and the expression levels of ZO-1 were determined by immunofluorescence staining **(D)** in the mouse colon. **(E)** Protein expression levels of TNFα, IL1β, and IL-6 were assessed by western blot analysis in mouse colon tissue. Data are presented as the mean ± SEM (*n* = 3). Statistically significant differences are indicated; *p < 0.05, **p < 0.01, ***p < 0.001, significantly different from control group.

The length of the colon is a key indicator of colon health. We found that after cold exposure, the length of the colon had a tendency to shorten, but no significant differences in length were observed (Figure 1A). However, H&E staining revealed that the colonic folds disappeared after cold exposure (Figure 1B). Significant decreases in the protein expression levels of the tight junction markers claudin-1, occludin and ZO-1 were observed after cold exposure, consistent with the ZO-1 immunofluorescence staining data (Figures 1C,D). Since increased inflammatory cytokine expression is a precursor of inflammation, we examined the effect of cold exposure on the expression of pro-inflammatory cytokines such as TNF-α, IL-6 and IL-1β. We found that cold exposure led to increased protein expression of pro-inflammatory cytokines *in vivo* (Figure 1E).

### Cold Exposure Induces Endoplasmic Reticulum Stress and Apoptosis in Mice Colon

**FIGURE 2:**
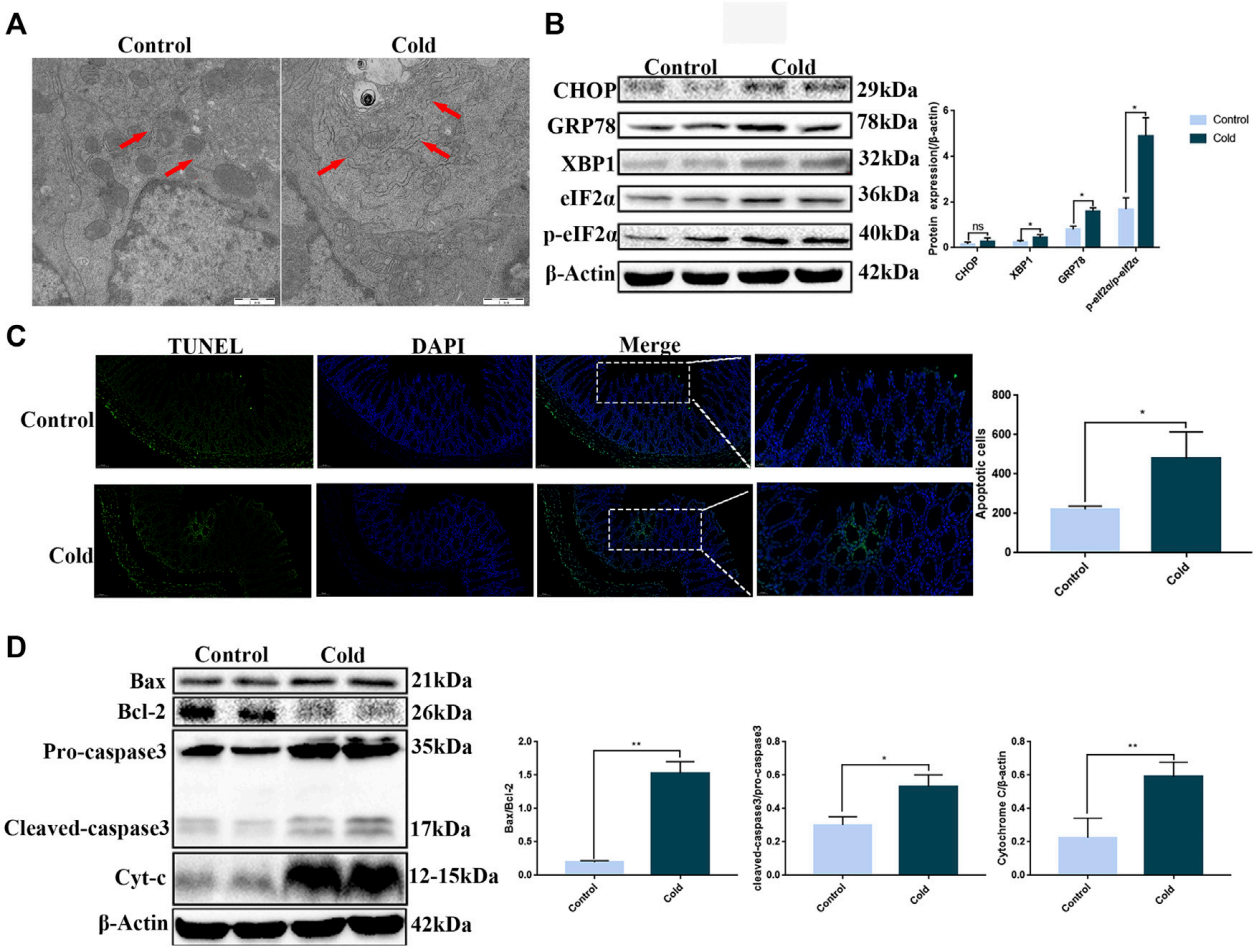
Cold exposure induces ER stress and apoptosis in mouse colon tissue. **(A)** Representative TEM images of mice colon, the red arrows indicate ER, scale bar: 1 µm. **(B)** CHOP, GRP78, XBP1, eIF2α, and p-eIF2α protein expression levels were measured by western blot analysis in mouse colon tissue. **(C)** Apoptosis was measured in mouse colon tissue using TUNEL staining. **(D)** Bax, Bcl-2, caspase-3, and Cytochrome C protein expression levels were measured by western blot analysis in mouse colon tissue. Data are presented as the mean ± SEM (*n* = 3). Statistically significant differences are indicated; *p < 0.05, **p < 0.01, ***p < 0.001, significantly different from control group.

Changes in the ultrastructure of the colon tissue were visualized by TEM. The ER was found to be swollen after cold exposure (Figure 2A). Since ER swelling may be caused by ER stress, we next used western blotting to examine protein expression of key proteins associated with ER stress. With the exception of CHOP, we found significant upregulation of GRP78, XBP1, eIF2α, and p-eIF2α protein expression in the cold exposure group (Figure 2B), suggesting that exposure to cold conditions induced ER stress in mice colon. ER stress, when dysregulated, can induce apoptosis. Therefore, we used TUNEL staining to examine apoptosis in the colonic epithelium of the mice. We found a significant increase in the number of apoptotic cells in the colonic tissue of the cold exposure group (Figure 2C). In order to investigate the correlation between the increase in apoptosis and ER stress, we next used western blotting to examine the expression of apoptotic proteins in the ER stress pathway, which are also key components of the endogenous apoptotic pathway. We found that Bax, Bcl-2, cleaved-caspase3 and Cytochrome C were significantly increased after cold exposure (Figure 2D). Thus, cold exposure results in increased ER stress and apoptosis in the colon.

### Cold Exposure Induces Oxidative Stress, Activates the Nrf2 Pathway, and Increases NF-κB and Nrf2 Acetylation Levels by Inhibiting SIRT1 in Mice Colon

**FIGURE 3:**
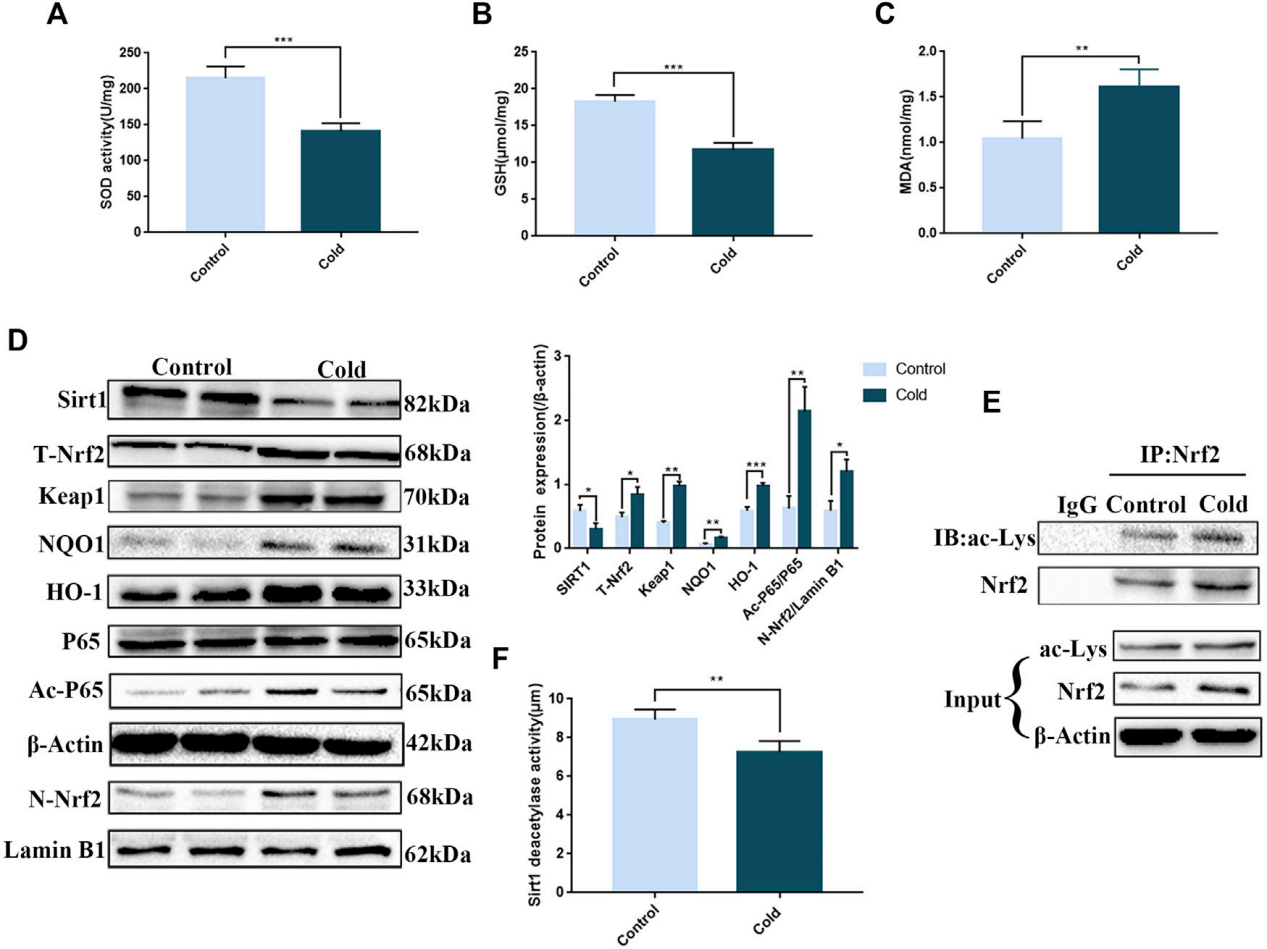
Cold exposure induces oxidative stress in the colon and regulates SIRT1/Nrf2-and SIRT1/NF-κB-mediated pathways *in vivo*. Effects of cold exposure on SOD **(A)**, GSH **(B)** and MDA **(C)** levels in the mouse colon. The protein expression levels **(D)** and enzyme activity **(F)** of SIRT1. **(D)** The protein expression levels of total-Nrf2,Keap1, nuclear-Nrf2, NQO1, HO-1, p65, and acetylated-p65 were measured by western blot analysis in mouse colon tissue. **(E)** The acetylation level of Nrf2 was determined using co-immunoprecipitation assays in mouse colon tissue. Data are presented as the mean ± SEM (*n* = 3). Statistically significant differences are indicated; *p < 0.05, **p < 0.01, ***p < 0.001, significantly different from control group.

Since ER stress is closely associated with oxidative stress, we examined the expression of oxidative stress markers in the colon. We found that SOD and GSH levels were significantly decreased, while MDA levels were significantly increased (Figures 3A–C), demonstrating that cold exposure induced oxidative stress in the colon. Many studies have shown that SIRT1 can act on antioxidant enzymes and has a role in mediating anti-oxidative stress (Hajializadeh and Khaksari, 2021). Here, we found that cold exposure significantly reduced SIRT1 protein expression and enzyme activity (Figure 3F), consistent with our data demonstrating that cold exposure enhanced oxidative stress *in vivo*. Cold exposure also activated the key antioxidant Nrf2 pathway, increased nuclear accumulation of Nrf2 and increased p65 protein expression to activate the inflammatory response (Figure 3D). Since SIRT1 was significantly reduced after cold exposure, we next used western blot and co-immunoprecipitation assays to examine acetylation of p65 and Nrf2. We found that cold exposure significantly increased p65 and Nrf2 acetylation (Figures 3D,E).

### Knock-Down of SIRT1 by shRNA Aggravates Tg-Induced Endoplasmic Reticulum Stress and Intercellular Tight Junction Damage, and Increases Apoptosis in Caco-2 CellsLife Science Identifiers

**FIGURE 4:**
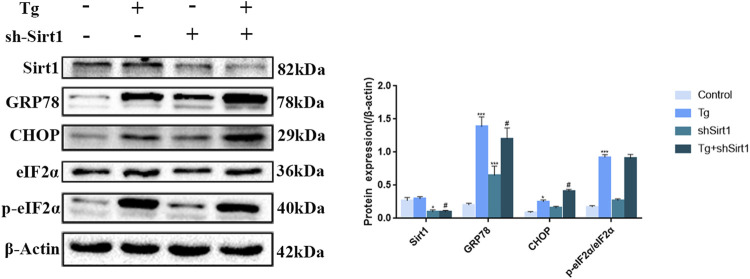
Effects of sh-SIRT1 on the protein levels of CHOP, GRP78, eIF2α and p-eIF2α in Tg-induced Caco-2 cells. Data are presented as the mean ± SEM (*n* = 3). Statistically significant differences are indicated; *p < 0.05, **p < 0.01, ***p < 0.001, significantly different from control group. #*p* < 0.05, significantly different from Tg group.

We next sought to determine the role of SIRT1 in mediating cold exposure-induced changes. Tg was used to induce ER stress in Caco-2 cells, and the effects of SIRT1 knock-down with shRNA on apoptosis and intercellular tight junctions were examined. We found that the expression of key proteins in the UPR pathway was significantly upregulated (Figure 4), indicating that a successful ER stress model had been established in Caco-2 cells, At the same time, the expression of SIRT1 in the sh-SIRT1 and Tg + sh-SIRT1 groups was significantly decreased (Figure 4), indicating that knock-down of SIRT1 with sh-SIRT1 was efficient. Consistent with previous studies (Alshammari et al., 2021), we demonstrated that SIRT1 has an inhibitory effect on ER stress since knock-down of SIRT1 resulted in a significant increase in GRP78 and CHOP protein expression (Figure 4).

**FIGURE 5:**
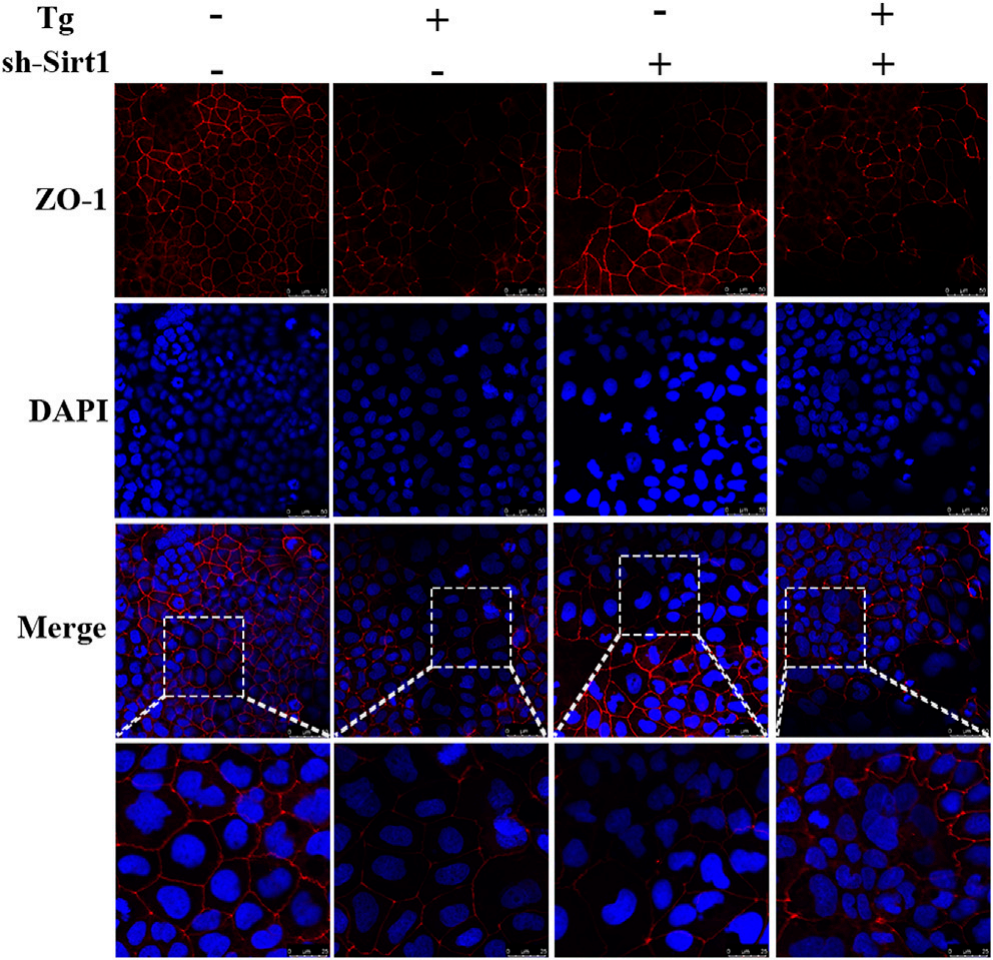
Effects of sh-SIRT1 on ZO-1 levels based on immunofluorescence staining of Tg-induced Caco-2 and IEC-6 cells. Data are presented as the mean ± SEM (*n* = 3). Statistically significant differences are indicated; *p < 0.05, **p < 0.01, ***p < 0.001, significantly different from control group. #*p* < 0.05, significantly different from Tg group.

Next, we visualized ZO-1 protein expression in Caco-2 cells by immunofluorescence analysis. ZO-1 distribution in the control group was uniform and complete, while in the Tg and sh-SIRT1 groups the distribution was uniform but the fluorescent intensity was lower than the control group. After treatment with Tg + sh-SIRT1, the expression level of ZO-1 in Caco-2 cells was significantly decreased, with some areas exhibiting no ZO-1 staining (Figure 5).

**FIGURE 6:**
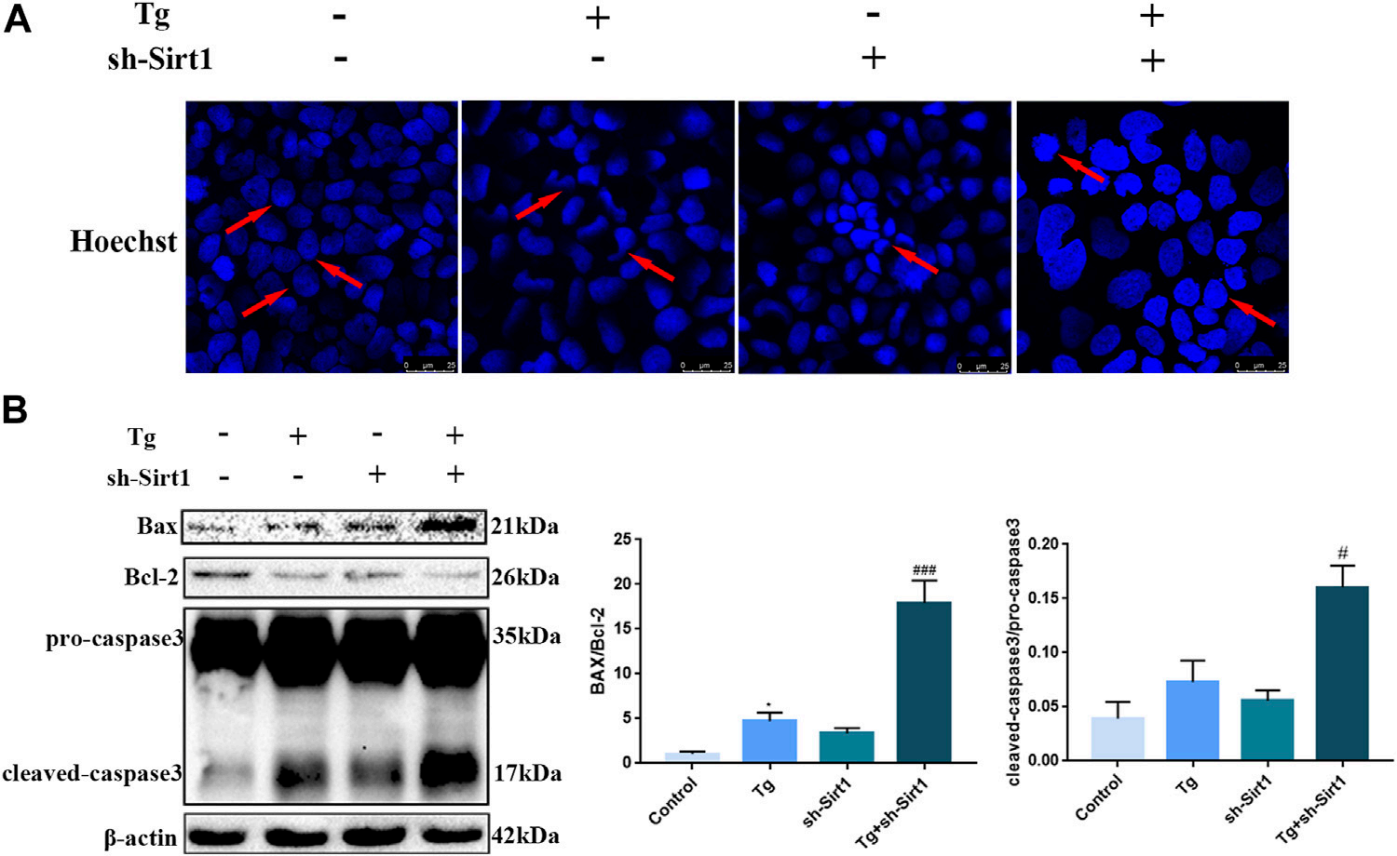
The knock-down of SIRT1 by shRNA increases the level of apoptosis in Tg-induced Caco-2 cells. **(A)** Apoptosis was measured in Tg-induced Caco-2 cells using Hoechst staining. **(B)** Effects of sh-SIRT1 on Bax, Bcl-2 and caspase-3 protein expression levels in Tg-induced Caco-2 cells. Data are presented as the mean ± SEM (*n* = 3). Statistically significant differences are indicated; *p < 0.05, **p < 0.01, ***p < 0.001, significantly different from control group. #*p* < 0.05, ##*p* < 0.01, ###*p* < 0.001 significantly different from Tg group.

Hoechst staining was used to examine apoptosis in Caco-2 cells. We found regular nuclear morphology and an even distribution of staining in the control group, while in the Tg and sh-SIRT1 groups slight nuclear deformation and intensive staining was observed. Treatment of Caco-2 cells with Tg + sh-SIRT1 resulted in a large area of concentrated staining, with visible nuclear fusion and deformation (Figure 6A). Changes in the expression levels of apoptotic proteins were also measured, and found to be consistent with the Hoechst staining data. Significant increases in the Bax/Bcl-2 protein expression ratio and cleaved-caspase-3 protein expression were observed in the Tg + sh-SIRT1 group (Figure 6B). Taken together, these data suggest that knock-down of SIRT1 aggravated Tg-induced tight junction damage and induced apoptosis in Caco-2 cells.

### Knock-Down of SIRT1 by shRNA Increases Nrf2 Nuclear Accumulation and Acetylation in Tg-Induced Caco-2 Cells

**FIGURE 7:**
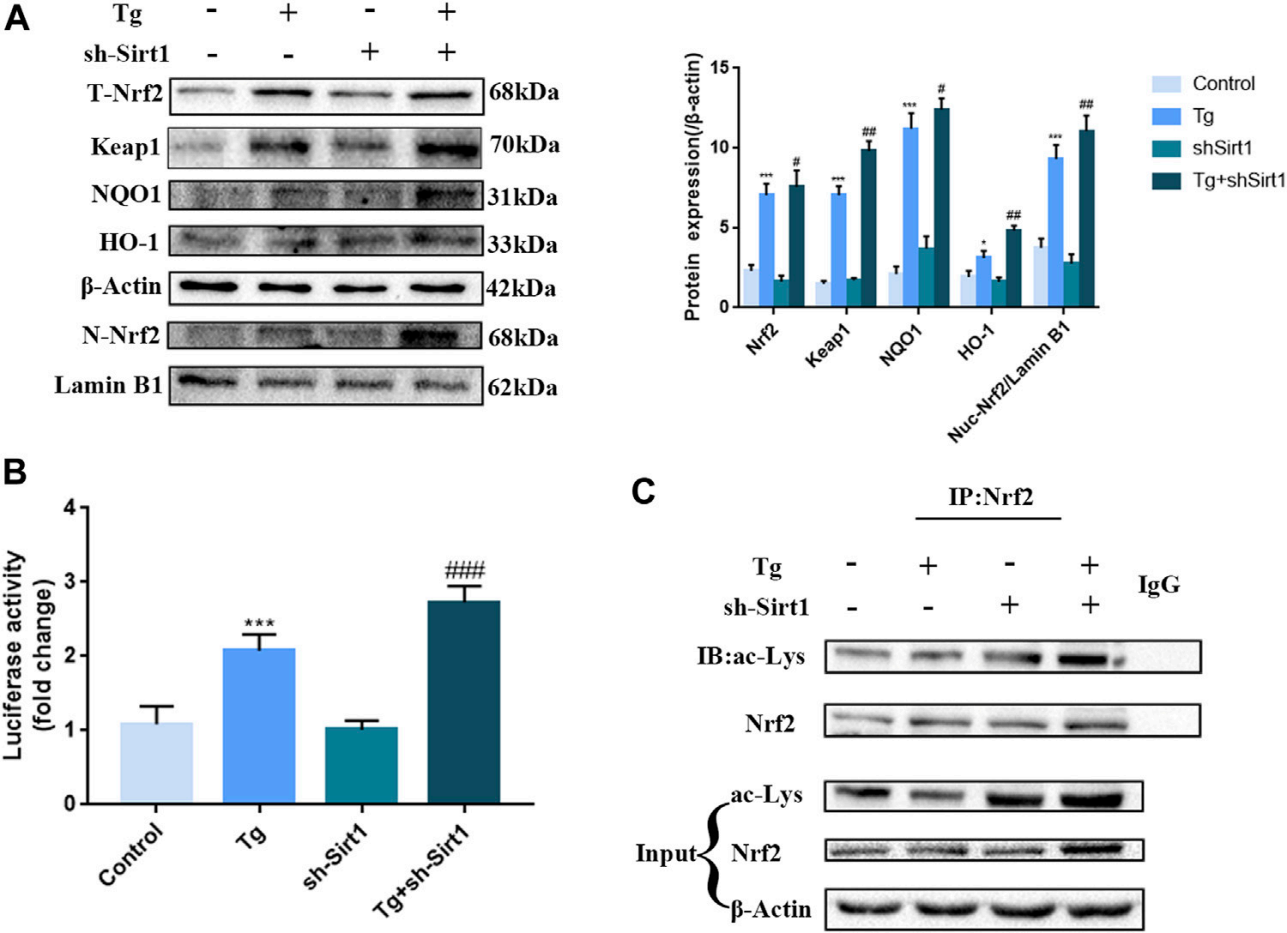
The knock-down of SIRT1 by shRNA increases Nrf2 nuclear accumulation and acetylation in Tg-induced Caco-2 cells. **(A)** Effects of sh-SIRT1 on total-Nrf2, Keap1,nuclear-Nrf2, NQO1 and HO-1 protein expression levels in Tg-induced Caco-2 cells were assessed by western blot analysis. **(B)** Effects of sh-SIRT1 on the relative fluorescence intensity of NRF2/ARE in Tg-induced Caco-2 cells were measured using the Dual Luciferase Reporter Gene Assay Kit. **(C)** Effects of sh-SIRT1 on Nrf2 acetylation levels were determined in Tg-induced Caco-2 cells by co-immunoprecipitation assays. Data are presented as the mean ± SEM (*n* = 3). Statistically significant differences are indicated; *p < 0.05, **p < 0.01, ***p < 0.001, significantly different from control group. #*p* < 0.05, ##*p* < 0.01, ###*p* < 0.001 significantly different from Tg group.

Next, we examined the role of SIRT1 in the Nrf2 pathway in Caco-2 cells. We found that in the Tg group, the protein expression of total Nrf2, nuclear Nrf2, Keap1and downstream components of the Nrf2/ARE signaling pathway, HO-1 and NQO1 was significantly up-regulated (Figure 7A), indicating that ER stress activated the antioxidant pathway in Caco-2 cells, promoted nuclear accumulation of Nrf2, and increased the transcriptional activity of Nrf2 (Figure 7B). In the Tg + sh-SIRT1 group, although no significant changes in total Nrf2 protein expression were observed, nuclear Nrf2 expression was significantly up-regulated. Furthermore, significant up-regulation of HO-1 and NQO1 was observed (Figure 6A), as well as significantly up-regulated transcriptional activity (Figure 7B). Finally, co-immunoprecipitation assays revealed that knock-down of SIRT1 led to significant up-regulation of Nrf2 acetylation (Figure 7C).

## Discussion

In the current study, we demonstrated that chronic cold exposure activated ER stress, which mediates apoptosis, inflammation and oxidative stress in the colon via the SIRT1/Nrf2 signaling pathway. Cold exposure activates the hypothalamic-pituitary-adrenal (HPA) axis, inducing a state of stress in the body (Schwabe et al., 2008). Since the colon has a large contact area with the external environment, it is a central target organ of environmental stress. Colonic length is an important indicator of colon health. Colonic shortening occurs in various enteritis such as IBD and ulcerative colitis (UC) (Otagiri et al., 2020; Wu et al., 2020). Here, no significant colonic shortening, or pathological damage was observed in response to cold exposure. However, H&E staining did reveal a phenomenon of wrinkle disappearance, suggesting that the effects of cold exposure on the colon were physiological, not pathological.

The colon acts as a multi-layer barrier to maintain colon homeostasis (Hamer et al., 2008). However, due to the abundance of colon flora, damage to the barrier may cause leakage of harmful bacteria and their metabolites, which contribute to the occurrence and development of colonic inflammation. In this process, the mechanical barrier, which is composed of tight junctions between cells, is crucial. The tight junction is composed of several transmembrane proteins and cytoplasmic proteins, including occludins, claudins, and ZOs (Otani and Furuse, 2020). Here, we found that ZO-1 protein expression was significantly down-regulated after cold exposure. ZO-1 is a cytoskeleton protein, which interacts with periplasmic membrane proteins including occludin, claudin and junction adhesion molecule (JAM) to form strong cross-links, as well as interacting with the membrane cytoskeleton composed of F-actin and myosin to maintain the integrity of barrier function (Haas et al., 2020). We found that the expression of occludin and claudin was also sharply down-regulated after cold exposure. Therefore, the breakdown of ZO-1, claudin-1 and occludin in cold-exposed mouse IECs can significantly affect the function of the colonic epithelial barrier.

Dysfunction of the colonic epithelial barrier can also promote the occurrence and development of colonic inflammation, with pro-inflammatory cytokines such as IL-1β, IL-6 and TNF-α playing a key role in the development of inflammation (Marafini et al., 2019). IL-1β and IL-6 are the main pro-inflammatory cytokines secreted by the early inflammatory response, which can initiate the release of other cytokines (Zhao et al., 2021). Zhang et al. found that the concentration of TNFα, IL-6 and IL-1β in lung tissue was significantly increased following acute cold stress (−25°C) (Zhang et al., 2015). Bal et al. found that FGF21, IL6, IL-1α and TNFα were significantly up-regulated in the serum of mice under chronic cold stress, similar to the findings from our acute cold stress model test (Bal et al., 2017). In our study, the pro-inflammatory cytokines IL-1β, IL-6 and TNF-α were significantly upregulated *in vivo* after cold exposure, consistent with previous studies, indicating that cold exposure could promote the occurrence of colonic inflammation.

Studies have shown that cold stress can significantly increase Hsp70 resulting in their binding to white blood cells followed by a rapid influx of calcium ions, while the maintenance of calcium homeostasis mainly depends on the ER (Tupling et al., 2008). IECs are metabolically active cells, which are sensitive to changes in protein folding homeostasis in the ER (Luo and Cao, 2015). Using TEM, we observed significant swelling of the ER in the cold exposure group, suggesting that cold exposure had induced ER stress.

ER stress is a precise cellular adaptive response to the accumulation of misfolded or unfolded proteins in the ER induced by external factors. Thus, we used western blot analysis to examine the expression levels of key proteins in the ER stress pathway in the mouse colon. We found that the expression levels of GRP78, XBP1, and p-eIF2α/eIF2α were significantly up-regulated after exposure to cold. In the healthy ER, Grp78 is a molecular chaperone that stably binds to PERK, IRE1α and ATF6 and maintains them in an inactive state. When cells encounter environmental challenges, the homeostasis of the ER is threatened. Accumulation of misfolded or unfolded proteins in the ER induces the dissociation of GRP78 from PERK, ATF6 and IRE1α and activates downstream signals, triggering UPR as a cellular adaptive response (Senft and Ronai, 2015). We found that cold exposure can promote GRP78 dissociation, and serine phosphorylation of eIF2α, resulting in inhibition of protein synthesis and promotion of cell survival. However, although PERK mediates cell survival, it is also setting the stage for cellular demise by establishing the temporal onset of CHOP accumulation. We also found significant up-regulation of CHOP protein expression in the cold exposure group. CHOP is a C/EBP homologous protein, also known as proapoptotic factor, which is considered to be the late initiation of UPR-activated PERK-eIF-2α axis (Chen et al., 2020). CHOP can directly regulate apoptosis factors, such as Bcl-2 and Bim, which subsequently make cells more prone to apoptosis (Chitnis et al., 2012). We found that apoptosis levels were significantly up-regulated after cold exposure both by TUNEL staining and by significant up-regulation of the Bcl2/Bax ratio, and Cytochrome C and cleaved-caspase-3 levels. Our findings indicated that the ER stress activated by cold exposure had entered the middle and late stages, and induced apoptosis.

Since many studies have reported a close connection between ER stress and oxidative stress (Feng et al., 2019; Zhao et al., 2020), we next examined MDA levels in the mouse colon. We found that MDA levels were significantly increased in the cold exposure group. MDA is the product of lipid peroxidation of polyunsaturated fatty acids, and is used to evaluate the degree of lipid peroxidation in tissues (Chen et al., 2013). As a marker of oxidative stress, up-regulation of MDA levels indicates that cold exposure leads to oxidative stress. At the same time, we found that expression of GSH and SOD, which play an important role in the antioxidant defense, decreased sharply after cold exposure. Thus, cold exposure induces oxidative stress *in vivo*.

SIRT1 is a NAD + -dependent histone deacetylase, which has been shown to play a major role in biological processes such as inflammation, apoptosis and oxidative stress (Tang, 2016). Therefore, we examined SIRT1 expression in mouse colon tissue, and found significant down-regulation of SIRT1 protein expression and enzyme activity in cold-exposed mice, indicating that cold exposure inhibited the activity and expression of SIRT1 in colon tissue. Recent studies have demonstrated that cold exposure mediates the cold stress response of mice. Therefore, due to the abnormal activation of the HPA axis, numerous GCs are released into the central nervous system upward through the blood-brain barrier in this process. The long-term accumulation of GCs will exert an influence on the expression of SIRT1 in microglia (Xu et al., 2020). However, the mechanism of cold exposure reducing the expression of sirt1 in the intestine is currently unknown and needs further exploration in subsequent studies. Our results showed that the acetylation level of p65 subunit lysine 310 was increased. Studies have shown that sirt1 can participate in regulating inflammatory responses by deacetylating the NFkb, and the NFkb p65 subunit at multiple lysine sites (Yeung et al., 2004). This modification will enhance the transcriptional activity of NF-kB and promote the binding of NFkb to its downstream pro-inflammatory factor promoter region (Yoshizaki et al., 2010). This finding is consistent with the results of high expression of the pro-inflammatory factors. In addition to regulating various biological processes *in vivo*, SIRT1 is also involved in the activation of transcription factor redox homeostasis, such as Nrf2 (Xu et al., 2021). Nrf2 plays a crucial role in regulating the expression and synergistic induction of a series of antioxidant phase 2 genes, and protecting cells from the cumulative damage of oxidative stress (Duranti et al., 2021). To date, the protective effect of Nrf2 in the colon has been confirmed by a large number of studies (Hybertson et al., 2011; Singh et al., 2019; Zhang et al., 2020b). Therefore, we examined Nrf2 expression in the mouse colon. In the normal mice, Nrf2 and Keap1 form a Keap1-Nrf2 complex. However, in the cold exposure group, We found that Nrf2 is released from the Keap1-Nrf2 complex, making Nrf2 easier to translocate to the nucleus, thereby up-regulating the expression of downstream antioxidant components of the Nrf2/ARE pathway HO-1 and NQO1. We also found that the acetylation level of Nrf2 was significantly increased after cold exposure, which may be related to the downregulation of SIRT1.

In order to confirm the role of SIRT1 in a series of changes induced by cold exposure, we established an ER stress model in Caco-2 cells using Tg to simulate cold exposure, and knocked down SIRT1 using shRNA. By measuring expression of key proteins of the ER stress pathway, we demonstrated that Tg successfully activated ER stress in Caco-2 cells, while SIRT1 knock-down increased the ER stress intensity. Recently, Liu et al. found that spermidine could inhibit ER stress by activating SIRT1, indicating that SIRT1 could inhibit Tg-induced ER stress *in vitro* (Liu et al., 2021). These findings were consistent with our study. We also found that knock-down of SIRT1 led to a significant decrease in ZO-1 immunofluorescence staining in Caco-2 cells, consistent with our *in vivo* data. In addition, we demonstrated that knock-down of SIRT1 increased cell death caused by Tg-induced ER stress, indicating that SIRT1 protected IECs from severe ER stress.

Based on our *in vivo* data, we speculate that cold exposure can increase the acetylation level of Nrf2 by reducing the expression and activity of SIRT1, thereby increasing Nrf2-dependent gene transcription. To verify this hypothesis, we examined the expression of Nrf2 and its downstream factors in Tg-treated Caco-2 cells. We found that Tg-induced ER stress up-regulated the protein expression of Nrf2 and its downstream factors, which might be due to the fact that the PERK pathway of ER stress could induce ATF4 and Nrf2. These two transcription factors could activate antioxidant stress response genes, including NQO1, HO-1 and glutathione transferase. Previous studies have found that the protection provided by SIRT1 occurs by regulating the main endogenous antioxidant system of the Nrf2/HO-1 pathway (Shi et al., 2019). It has been reported that Nrf2 is an important antioxidant sensor in cell defense mechanism, which can be used as an important downstream target for SIRT1 signal transduction to increase the resistance to oxidative damage (Zhou et al., 2015). Our study concluded that the knockdown of SIRT1 led to increased Nrf2 acetylation, and a subsequent increase in Nrf2 nuclear localization and transcriptional activity. Studies have also shown that SIRT1 can induce NRF2 deacetylation, thereby reducing NRF2 - dependent gene transcription (Simic et al., 2013). Therefore, increased nrf2 acetylation induced by sirt1 knockdown increases nrf2-dependent gene transcription to resist the colonic oxidative stress induced by ER stress.

## Conclusion

In conclusion, our study demonstrated that cold exposure can lead to unfolded or misfolded protein accumulation in colonic ER, and subsequent induction of ER stress in the colon. Excessive activation of ER stress can promote cell apoptosis, activating Nrf2 pathway, as well as activate nuclear accumulation of the antioxidant factor Nrf2, increase its transcriptional activity, and activate the downstream antioxidant factors HO-1 and NQO1. In this process, the decrease in SIRT1 expression and activity has a two-way effect that intensifies ER stress and increases the acetylation level of Nrf2, leading to the nuclear accumulation of Nrf2. Therefore, SIRT1 can be used as a potential target for alleviating colonic injury caused by cold exposure.

## Data Availability

The original contributions presented in the study are included in the article/Supplementary Material, further inquiries can be directed to the corresponding authors.

## References

[B1] Almenier H. A. Al Menshawy H. H. Maher M. M. Al Gamal S. (2012). Oxidative Stress and Inflammatory Bowel Disease. Front. Biosci. E4, 1335–1344. 10.2741/463 10.2741/463 | Google Scholar 22201958

[B2] Alshammari G. M. Al-Qahtani W. H. AlFaris N. A. Albekairi N. A. Alqahtani S. Eid R. (2021). Quercetin Alleviates Cadmium Chloride-Induced Renal Damage in Rats by Suppressing Endoplasmic Reticulum Stress through SIRT1-dependent Deacetylation of Xbp-1s and eIF2α. Biomed. Pharmacother. 141, 111862. 10.1016/j.biopha.2021.111862 PubMed Abstract | 10.1016/j.biopha.2021.111862 | Google Scholar 34246189

[B3] Bal N. C. Maurya S. K. Pani S. Sethy C. Banerjee A. Das S. (2017). Mild Cold Induced Thermogenesis: Are BAT and Skeletal Muscle Synergistic Partners? Biosci. Rep. 37, BSR20171087. 10.1042/BSR20171087 PubMed Abstract | 10.1042/BSR20171087 | Google Scholar 28831023 PMC5617911

[B4] Bandyopadhayaya S. Ford B. Mandal C. C. (2020). Cold-Hearted: A Case for Cold Stress in Cancer Risk. J. Therm. Biol. 91, 102608. 10.1016/j.jtherbio.2020.102608 PubMed Abstract | 10.1016/j.jtherbio.2020.102608 | Google Scholar 32716858

[B5] Chandramowlishwaran P. Vijay A. Abraham D. Li G. Mwangi S. M. Srinivasan S. (2020). Role of Sirtuins in Modulating Neurodegeneration of the Enteric Nervous System and Central Nervous System. Front. Neurosci. 14, 614331. 10.3389/fnins.2020.614331 PubMed Abstract | 10.3389/fnins.2020.614331 | Google Scholar 33414704 PMC7783311

[B6] Chen J. Zhuang T. Chen J. Tian Y. Yi X. Ni Q. (2020). Homocysteine Induces Melanocytes Apoptosis via PERK-eIF2α-CHOP Pathway in Vitiligo. Clin. Sci. (Lond). 134 (10), 1127–1141. 10.1042/CS20200218 PubMed Abstract | 10.1042/CS20200218 | Google Scholar 32400851

[B7] Chen X. Zhai X. Shi J. Liu W. W. Tao H. Sun X. (2013). Lactulose Mediates Suppression of Dextran Sodium Sulfate-Induced colon Inflammation by Increasing Hydrogen Production. Dig. Dis. Sci. 58 (6), 1560–1568. 10.1007/s10620-013-2563-7 PubMed Abstract | 10.1007/s10620-013-2563-7 | Google Scholar 23371012

[B8] Chitnis N. S. Pytel D. Bobrovnikova-Marjon E. Pant D. Zheng H. Maas N. L. (2012). miR-211 Is a Prosurvival microRNA that Regulates Chop Expression in a PERK-dependent Manner. Mol. Cell. 48 (3), 353–364. 10.1016/j.molcel.2012.08.025 PubMed Abstract | 10.1016/j.molcel.2012.08.025 | Google Scholar 23022383 PMC3496065

[B9] Chou X. Ding F. Zhang X. Ding X. Gao H. Wu Q. (2019). Sirtuin-1 Ameliorates Cadmium-Induced Endoplasmic Reticulum Stress and Pyroptosis through XBP-1s Deacetylation in Human Renal Tubular Epithelial Cells. Arch. Toxicol. 93 (4), 965–986. 10.1007/s00204-019-02415-8 PubMed Abstract | 10.1007/s00204-019-02415-8 | Google Scholar 30796460

[B10] Cullinan S. B. Diehl J. A. (2006). Coordination of ER and Oxidative Stress Signaling: the PERK/Nrf2 Signaling Pathway. Int. J. Biochem. Cell Biol. 38 (3), 317–332. 10.1016/j.biocel.2005.09.018 PubMed Abstract | 10.1016/j.biocel.2005.09.018 | Google Scholar 16290097

[B11] Deuring J. J. Fuhler G. M. Konstantinov S. R. Peppelenbosch M. P. Kuipers E. J. de Haar C. (2014). Genomic ATG16L1 Risk Allele-Restricted Paneth Cell ER Stress in Quiescent Crohn's Disease. Gut. 63 (7), 1081–1091. 10.1136/gutjnl-2012-303527 PubMed Abstract | 10.1136/gutjnl-2012-303527 | Google Scholar 23964099

[B12] Duranti G. Maldini M. Crognale D. Horner K. Dimauro I. Sabatini S. (2021). Moringa Oleifera Leaf Extract Upregulates Nrf2/HO-1 Expression and Ameliorates Redox Status in C2C12 Skeletal Muscle Cells. Molecules. 26 (16), 5041. 10.3390/molecules26165041 PubMed Abstract | 10.3390/molecules26165041 | Google Scholar 34443628 PMC8400669

[B13] Evans C. A. Corfe B. M. (2021). Colorectal Keratins: Integrating Nutrition, Metabolism and Colorectal Health. Semin. Cell Developmental Biol. (21), S108400222–S108495216. 10.1016/j.semcdb.2021.08.010 10.1016/j.semcdb.2021.08.010 | Google Scholar 34481710

[B14] Feng K. Chen Z. Pengcheng L. Zhang S. Wang X. (2019). Quercetin Attenuates Oxidative Stress‐induced Apoptosis via SIRT1/AMPK‐mediated Inhibition of ER Stress in Rat Chondrocytes and Prevents the Progression of Osteoarthritis in a Rat Model. J. Cell Physiol. 234 (10), 18192–18205. 10.1002/jcp.28452 PubMed Abstract | 10.1002/jcp.28452 | Google Scholar 30854676

[B15] Haas A. J. Zihni C. Ruppel A. Hartmann C. Ebnet K. Tada M. (2020). Interplay between Extracellular Matrix Stiffness and JAM-A Regulates Mechanical Load on ZO-1 and Tight Junction Assembly. Cell Rep. 32 (3), 107924. 10.1016/j.celrep.2020.107924 PubMed Abstract | 10.1016/j.celrep.2020.107924 | Google Scholar 32697990 PMC7383227

[B16] Hajializadeh Z. Khaksari M. (2021). The Protective Effects of 17-β Estradiol and SIRT1 against Cardiac Hypertrophy: a Review. Heart Fail. Rev. 27, 725–738. 10.1007/s10741-021-10171-0 PubMed Abstract | 10.1007/s10741-021-10171-0 | Google Scholar 34537933

[B17] Hamer H. M. Jonkers D. Venema K. Vanhoutvin S. Troost F. J. Brummer R.-J. (2008). Review Article: the Role of Butyrate on Colonic Function. Aliment. Pharmacol. Ther. 27 (2), 104–119. 10.1111/j.1365-2036.2007.03562.x PubMed Abstract | 10.1111/j.1365-2036.2007.03562.x | Google Scholar 17973645

[B18] Hosomi S. Kaser A. Blumberg R. S. (2015). Role of Endoplasmic Reticulum Stress and Autophagy as Interlinking Pathways in the Pathogenesis of Inflammatory Bowel Disease. Curr. Opin. Gastroenterol. 31 (1), 81–88. 10.1097/MOG.0000000000000144 PubMed Abstract | 10.1097/MOG.0000000000000144 | Google Scholar 25426970 PMC4592163

[B19] Hybertson B. M. Gao B. Bose S. K. McCord J. M. (2011). Oxidative Stress in Health and Disease: the Therapeutic Potential of Nrf2 Activation. Mol. Aspects Med. 32 (4-6), 234–246. 10.1016/j.mam.2011.10.006 PubMed Abstract | 10.1016/j.mam.2011.10.006 | Google Scholar 22020111

[B20] Kaser A. Adolph T. E. Blumberg R. S. (2013). The Unfolded Protein Response and Gastrointestinal Disease. Semin. Immunopathol 35 (3), 307–319. 10.1007/s00281-013-0377-5 PubMed Abstract | 10.1007/s00281-013-0377-5 | Google Scholar 23588234 PMC3661271

[B21] Li X.-X. Zhang H.-S. Xu Y.-M. Zhang R.-J. Chen Y. Fan L. (2017). Knockdown of IRE1α Inhibits Colonic Tumorigenesis through Decreasing β-catenin and IRE1α Targeting Suppresses colon Cancer Cells. Oncogene. 36 (48), 6738–6746. 10.1038/onc.2017.284 PubMed Abstract | 10.1038/onc.2017.284 | Google Scholar 28825721

[B22] Lian S. Xu B. Wang D. Wang L. Li W. Yao R. (2019). Possible Mechanisms of Prenatal Cold Stress Induced-anxiety-like Behavior Depression in Offspring Rats. Behav. Brain Res. 359, 304–311. 10.1016/j.bbr.2018.11.008 PubMed Abstract | 10.1016/j.bbr.2018.11.008 | Google Scholar 30423388

[B23] Liu X. Chen A. Liang Q. Yang X. Dong Q. Fu M. (2021). Spermidine Inhibits Vascular Calcification in Chronic Kidney Disease through Modulation of SIRT1 Signaling Pathway. Aging Cell. 20 (6), e13377. 10.1111/acel.13377 PubMed Abstract | 10.1111/acel.13377 | Google Scholar 33969611 PMC8208796

[B24] Luo K. Cao S. S. (2015). Endoplasmic Reticulum Stress in Intestinal Epithelial Cell Function and Inflammatory Bowel Disease. Gastroenterol. Res. Pract. 2015, 1–6. 10.1155/2015/328791 10.1155/2015/328791 | Google Scholar PMC4338396 25755668

[B25] Ma X. Dai Z. Sun K. Zhang Y. Chen J. Yang Y. (2017). Intestinal Epithelial Cell Endoplasmic Reticulum Stress and Inflammatory Bowel Disease Pathogenesis: An Update Review. Front. Immunol. 8, 1271. 10.3389/fimmu.2017.01271 PubMed Abstract | 10.3389/fimmu.2017.01271 | Google Scholar 29118753 PMC5660968

[B26] Malhotra J. D. Kaufman R. J. (2007). Endoplasmic Reticulum Stress and Oxidative Stress: a Vicious Cycle or a Double-Edged Sword? Antioxid. Redox Signaling. 9 (12), 2277–2294. 10.1089/ars.2007.1782 PubMed Abstract | 10.1089/ars.2007.1782 | Google Scholar 17979528

[B27] Marafini I. Sedda S. Dinallo V. Monteleone G. (2019). Inflammatory Cytokines: from Discoveries to Therapies in IBD. Expert Opin. Biol. Ther. 19 (11), 1207–1217. 10.1080/14712598.2019.1652267 PubMed Abstract | 10.1080/14712598.2019.1652267 | Google Scholar 31373244

[B28] Melhem H. Hansmannel F. Bressenot A. Battaglia-Hsu S.-F. Billioud V. Alberto J. M. (2016). Methyl-deficient Diet Promotes Colitis and SIRT1-Mediated Endoplasmic Reticulum Stress. Gut. 65 (4), 595–606. 10.1136/gutjnl-2014-307030 PubMed Abstract | 10.1136/gutjnl-2014-307030 | Google Scholar 25608526

[B29] Öhman L. Törnblom H. Simrén M. (2015). Crosstalk at the Mucosal Border: Importance of the Gut Microenvironment in IBS. Nat. Rev. Gastroenterol. Hepatol. 12 (1), 36–49. 10.1038/nrgastro.2014.200 PubMed Abstract | 10.1038/nrgastro.2014.200 | Google Scholar 25446728

[B30] Otagiri S. Ohnishi S. Ohara M. Fu Q. Yamamoto K. Yamamoto K. (2020). Oleoylethanolamide Ameliorates Dextran Sulfate Sodium-Induced Colitis in Rats. Front. Pharmacol. 11, 1277. 10.3389/fphar.2020.01277 PubMed Abstract | 10.3389/fphar.2020.01277 | Google Scholar 32922296 PMC7457075

[B31] Otani T. Furuse M. (2020). Tight Junction Structure and Function Revisited. Trends Cell Biol. 30 (10), 805–817. 10.1016/j.tcb.2020.08.004 PubMed Abstract | 10.1016/j.tcb.2020.08.004 | Google Scholar 32891490

[B32] Pellissier S. Bonaz B. (2017). The Place of Stress and Emotions in the Irritable Bowel Syndrome. Vitam Horm. 103, 327–354. 10.1016/bs.vh.2016.09.005 PubMed Abstract | 10.1016/bs.vh.2016.09.005 | Google Scholar 28061975

[B33] Prola A. Pires Da Silva J. Guilbert A. Lecru L. Piquereau J. Ribeiro M. (2017). SIRT1 Protects the Heart from ER Stress-Induced Cell Death through eIF2α Deacetylation. Cell Death Differ. 24 (2), 343–356. 10.1038/cdd.2016.138 PubMed Abstract | 10.1038/cdd.2016.138 | Google Scholar 27911441 PMC5299716

[B34] Schwabe L. Haddad L. Schachinger H. (2008). HPA axis Activation by a Socially Evaluated Cold-Pressor Test. Psychoneuroendocrinology. 33 (6), 890–895. 10.1016/j.psyneuen.2008.03.001 PubMed Abstract | 10.1016/j.psyneuen.2008.03.001 | Google Scholar 18403130

[B35] Senft D. Ronai Z. e. A. (2015). UPR, Autophagy, and Mitochondria Crosstalk Underlies the ER Stress Response. Trends Biochem. Sci. 40 (3), 141–148. 10.1016/j.tibs.2015.01.002 PubMed Abstract | 10.1016/j.tibs.2015.01.002 | Google Scholar 25656104 PMC4340752

[B36] Shi S. Lei S. Tang C. Wang K. Xia Z. (2019). Melatonin Attenuates Acute Kidney Ischemia/reperfusion Injury in Diabetic Rats by Activation of the SIRT1/Nrf2/HO-1 Signaling Pathway. Biosci. Rep. 39 (1), BSR20181614. 10.1042/BSR20181614 PubMed Abstract | 10.1042/BSR20181614 | Google Scholar 30578379 PMC6331666

[B37] Simic P. Zainabadi K. Bell E. Sykes D. B. Saez B. Lotinun S. (2013). SIRT1 Regulates Differentiation of Mesenchymal Stem Cells by Deacetylating β‐catenin. EMBO Mol. Med. 5 (3), 430–440. Epub 2013 Jan 30. Erratum in: EMBO Mol Med. 2013 Mar;5(3):482. 10.1002/emmm.201201606 PubMed Abstract | 10.1002/emmm.201201606 | Google Scholar 23364955 PMC3598082

[B38] Singh R. Chandrashekharappa S. Bodduluri S. R. Baby B. V. Hegde B. Kotla N. G. (2019). Enhancement of the Gut Barrier Integrity by a Microbial Metabolite through the Nrf2 Pathway. Nat. Commun. 10 (1), 89. 10.1038/s41467-018-07859-7 PubMed Abstract | 10.1038/s41467-018-07859-7 | Google Scholar 30626868 PMC6327034

[B39] Singh S. Nandi A. Banerjee O. Bhattacharjee A. Prasad S. K. Maji B. K. (2020). Cold Stress Modulates Redox Signalling in Murine Fresh Bone Marrow Cells and Promotes Osteoclast Transformation. Arch. Physiol. Biochem. 126 (4), 348–355. 10.1080/13813455.2018.1538249 PubMed Abstract | 10.1080/13813455.2018.1538249 | Google Scholar 30468085

[B40] Solà Tapias N. Denadai-Souza A. Rolland-Fourcade C. Quaranta-Nicaise M. Blanpied C. Marcellin M. (2021). Colitis Linked to Endoplasmic Reticulum Stress Induces Trypsin Activity Affecting Epithelial Functions. J. Crohns Colitis. 15 (9), 1528–1541. PMID: 33609354. 10.1093/ecco-jcc/jjab035 PubMed Abstract | 10.1093/ecco-jcc/jjab035 | Google Scholar 33609354

[B41] Tang B. L. (2016). Sirt1 and the Mitochondria. Mol. Cell. 39 (2), 87–95. 10.14348/molcells.2016.2318 PubMed Abstract | 10.14348/molcells.2016.2318 | Google Scholar PMC4757807 26831453

[B42] Tupling A. R. Bombardier E. Vigna C. Quadrilatero J. Fu M. (2008). Interaction between Hsp70 and the SR Ca2+pump: a Potential Mechanism for Cytoprotection in Heart and Skeletal Muscle. Appl. Physiol. Nutr. Metab. 33 (5), 1023–1032. 10.1139/H08-067 PubMed Abstract | 10.1139/H08-067 | Google Scholar 18923580

[B43] Wang D. Cheng X. Fang H. Ren Y. Li X. Ren W. (2020). Effect of Cold Stress on Ovarian & Uterine Microcirculation in Rats and the Role of Endothelin System. Reprod. Biol. Endocrinol. 18 (1), 29. 10.1186/s12958-020-00584-1 PubMed Abstract | 10.1186/s12958-020-00584-1 | Google Scholar 32290862 PMC7155299

[B44] Wang R. Moniruzzaman M. Wong K. Y. Wiid P. Harding A. Giri R. (2021). Gut Microbiota Shape the Inflammatory Response in Mice with an Epithelial Defect. Gut Microbes. 13 (1), 1–18. 10.1080/19490976.2021.1887720 10.1080/19490976.2021.1887720 | Google Scholar PMC7928202 33645438

[B45] Wu J. Wei Z. Cheng P. Qian C. Xu F. Yang Y. (2020). Rhein Modulates Host Purine Metabolism in Intestine through Gut Microbiota and Ameliorates Experimental Colitis. Theranostics. 10 (23), 10665–10679. 10.7150/thno.43528 PubMed Abstract | 10.7150/thno.43528 | Google Scholar 32929373 PMC7482825

[B46] Wu T. Yao H. Zhang B. Zhou S. Hou P. Chen K. (2021). κ Opioid Receptor Agonist Inhibits Myocardial Injury in Heart Failure Rats through Activating Nrf2/HO-1 Pathway and Regulating Ca2+-SERCA2a. Oxidative Med. Cell Longevity 2021, 1–13. 10.1155/2021/7328437 10.1155/2021/7328437 | Google Scholar PMC8349291 34373768

[B47] Xu B. Lang L. Lian S. Guo J.-R. Wang J.-F. Liu J. (2020). Neuroinflammation Induced by Secretion of Acetylated HMGB1 from Activated Microglia in Hippocampi of Mice Following Chronic Cold Exposure. Brain Res. 1726, 146495. 10.1016/j.brainres.2019.146495 PubMed Abstract | 10.1016/j.brainres.2019.146495 | Google Scholar 31586627

[B48] Xu B. Zang S. Li S. Guo J. Wang J. Wang D. (2019). HMGB1-mediated Differential Response on Hippocampal Neurotransmitter Disorder and Neuroinflammation in Adolescent Male and Female Mice Following Cold Exposure. Brain Behav. Immun. 76, 223–235. 10.1016/j.bbi.2018.11.313 PubMed Abstract | 10.1016/j.bbi.2018.11.313 | Google Scholar 30476565

[B49] Xu J.-J. Cui J. Lin Q. Chen X.-Y. Zhang J. Gao E.-H. (2021). Protection of the Enhanced Nrf2 Deacetylation and its Downstream Transcriptional Activity by SIRT1 in Myocardial Ischemia/reperfusion Injury. Int. J. Cardiol. 342, 82–93. 10.1016/j.ijcard.2021.08.007 PubMed Abstract | 10.1016/j.ijcard.2021.08.007 | Google Scholar 34403762

[B50] Yao R. Yang Y. Lian S. Shi H. Liu P. Liu Y. (2018). Effects of Acute Cold Stress on Liver O-GlcNAcylation and Glycometabolism in Mice. Int J Mol Sci. 19 (9), 2815. 10.3390/ijms19092815 PubMed Abstract | 10.3390/ijms19092815 | Google Scholar PMC6165085 30231545

[B51] Yeung F. Hoberg J. E. Ramsey C. S. Keller M. D. Jones D. R. Frye R. A. (2004). Modulation of NF-κB-dependent Transcription and Cell Survival by the SIRT1 Deacetylase. EMBO J. 23 (12), 2369–2380. 10.1038/sj.emboj.7600244 PubMed Abstract | 10.1038/sj.emboj.7600244 | Google Scholar 15152190 PMC423286

[B52] Yoshizaki T. Schenk S. Imamura T. Babendure J. L. Sonoda N. Bae E. J. (2010). SIRT1 Inhibits Inflammatory Pathways in Macrophages and Modulates Insulin Sensitivity. Am. J. Physiology-Endocrinology Metab. 298 (3), E419–E428. 10.1152/ajpendo.00417.2009 PubMed Abstract | 10.1152/ajpendo.00417.2009 | Google Scholar PMC2838524 19996381

[B53] Zhang Y. Li X. Zhang L. Lin Y. S. Xiao Z. H. Su Z. (2015). Effects of Acute Cold Exposure on Pulmonary Proinflammatory Cytokine of Rat. Zhongguo Ying Yong Sheng Li Xue Za Zhi. 31 (1), 6–9. PubMed Abstract | Google Scholar 26016226

[B54] Zhang Y. Wang S. Zhang X. Hu Q. Zheng C. (2020a). Association between Moderately Cold Temperature and Mortality in China. Environ. Sci. Pollut. Res. Int. 27 (10), 26211–26220. 10.1007/s11356-020-08960-5 PubMed Abstract | 10.1007/s11356-020-08960-5 | Google Scholar 32361971

[B55] Zhang Y. Yan T. Sun D. Xie C. Wang T. Liu X. (2020b). Rutaecarpine Inhibits KEAP1-NRF2 Interaction to Activate NRF2 and Ameliorate Dextran Sulfate Sodium-Induced Colitis. Free Radic. Biol. Med. 148, 33–41. 10.1016/j.freeradbiomed.2019.12.012 PubMed Abstract | 10.1016/j.freeradbiomed.2019.12.012 | Google Scholar 31874248 PMC7376370

[B56] Zhao N. Yang F. E. Zhao C. Y. Lv S. W. Wang J. Liu J. M. (2021). Construction of pH‐Dependent Nanozymes with Oxygen Vacancies as the High‐Efficient Reactive Oxygen Species Scavenger for Oral‐Administrated Anti‐Inflammatory Therapy. Adv. Healthc. Mater. 10, 2101618. 10.1002/adhm.202101618 10.1002/adhm.202101618 | Google Scholar 34569192

[B57] Zhao T. Wu K. Hogstrand C. Xu Y.-H. Chen G.-H. Wei C.-C. (2020). Lipophagy Mediated Carbohydrate-Induced Changes of Lipid Metabolism via Oxidative Stress, Endoplasmic Reticulum (ER) Stress and ChREBP/PPARγ Pathways. Cell. Mol. Life Sci. 77 (10), 1987–2003. 10.1007/s00018-019-03263-6 PubMed Abstract | 10.1007/s00018-019-03263-6 | Google Scholar 31392349 PMC11105093

[B58] Zhou L. Xu D.-y. Sha W.-g. Shen L. Lu G.-y. Yin X. (2015). High Glucose Induces Renal Tubular Epithelial Injury via Sirt1/NF-kappaB/microR-29/Keap1 Signal Pathway. J. Transl Med. 13, 352. 10.1186/s12967-015-0710-y PubMed Abstract | 10.1186/s12967-015-0710-y | Google Scholar 26552447 PMC4640239

